# CT vascular territory mapping: a novel method to identify large vessel occlusion collateral

**DOI:** 10.1007/s00234-022-03034-4

**Published:** 2022-08-11

**Authors:** Michael Valente, Andrew Bivard, Andrew Cheung, Nathan W. Manning, Mark W. Parsons

**Affiliations:** 1 Department of Medicine and Neurology, Melbourne Brain Centre at the Royal Melbourne Hospital, University of Melbourne, Parkville, Australia; 2 Neurointervention, Liverpool Hospital, Liverpool, NSW Australia; 3 Neurointervention, Prince of Wales Hospital, Randwick, NSW Australia; 4 Ingham Institute of Applied Medical Research, Liverpool, NSW Australia; 5 South West Sydney Clinical Campuses, University of New South Wales, Sydney, Australia; 6 Department of Neurology Liverpool Hospital, Ingham Institute of Applied Medical Research, University of New South Wales South West Sydney Clinical Campuses, Liverpool, Australia

**Keywords:** Territory mapping, Ischaemic stroke, Neuroradiology, CT perfusion, Collateral flow

## Abstract

**Introduction:**

This descriptive study explores typical patterns of vascular territory mapping (VTM) in ischaemic stroke patients with proximal vessel occlusion. VTM is a novel process using CT perfusion that can identify the source and extent of collateral blood flow in patients with vessel occlusion. It functions by determining which vessel provides dominant blood flow to a brain voxel.

**Methods:**

A total of 167 consecutive patients were analysed from INSPIRE (International Stroke Perfusion Imaging Registry) with their CT perfusion reprocessed through VTM software. We explored the typical territory maps generated by this software relating to common large vessel occlusion location sites (ACA/MCA/PCA).

**Results/Conclusion:**

In the presence of occlusion, VTM demonstrated a reciprocal increase in collateral vessel territories.

**Supplementary Information:**

The online version contains supplementary material available at 10.1007/s00234-022-03034-4.

## Introduction

VTM is a process that provides an automated assessment of individual vessel flow using CT perfusion. It is a novel process that has recently been described by Matsubara et al. [1] and Christensen S. et al. [2] The software is used to provide an estimate as to which large vessel (ACA/MCA/PCA) provides the predominant flow to a brain region. In the setting of arterial occlusion, VTM is a map that describes the predominant collateral flow to a brain region. The clinical utility of VTM in the setting of ischaemic stroke secondary to large vessel occlusion (LVO) has not been explored.

Determination of collateral flow in patients with ischaemic stroke is important since it is highly predictive of irreversible infarction [3]. A favourable collateral profile will indicate whether a patient can benefit from reperfusion therapies (thrombolysis and/or endovascular therapy). Collateral supply is highly variable and can originate from any of the cerebral arteries: anterior cerebral artery (ACA), middle cerebral artery (MCA), or posterior cerebral artery (PCA). Single or multiphase CTA can be used to provide vessel-specific information and has also been shown to reliably predict outcomes post revascularization [4]. This visual estimation process is often omitted since it is time consuming and subject to wide inter-observer variability [5]. Arterial spin labelling with MRI [6] can enable visualization of vascular arterial territories, however, it is often not feasible to perform MRI in the hyperacute stroke setting.

VTM can be easily incorporated into hyperacute stroke workflow since it is a software solution that provides supplementary information to the CT Perfusion (CTP). In many centres, the current stroke workflow includes non-contrast CT brain (NCCT), CTP, and CT arch to vertex angiogram (CTA). Although CTP has become the cornerstone of collateral assessment [3], the current CTP display lacks information regarding vessel-specific collateral contributions. The VTM does not require additional images to be performed and uses the time-to-peak (TTP) map and anatomical landmarks to generate a map of collateral contributions.

This descriptive paper aimed to examine the use of VTM in ischaemic stroke patients with proximal vessel occlusion. We describe the typical VTM generated by this software relating to common large vessel occlusion location sites (ACA/MCA/PCA).

## Materials and methods

Consecutive patients were analysed from INSPIRE (International Stroke Perfusion Imaging Registry) with their CTP reprocessed through VTM software. Participants required CT perfusion images to have been performed on Toshiba Aquilion One 320 Slice (Toshiba Medical Systems, Otawara, Japan) due to its superior whole brain coverage. Participants were excluded if CTP data was incomplete or if the VTM seeding algorithm failed to process. INSPIRE is a registry of ischaemic stroke patients presenting to the hospital within 12 h from symptom onset between 2010 and 2018. Contributing to the database were seven hospitals in Australia, Canada, and China. As part of INSPIRE, patients underwent baseline multimodal CT imaging with non-contrast CT, CTP, and follow-up imaging with magnetic resonance imaging 24 h poststroke. All patients within the INSPIRE study provided written consent for their data to be used.

After data processing was completed, the authors reviewed maps to identify typical trends and volumes based on LVO location. The following data was collated for each patient: demographic information, VTM output volumes, collateral index, acute CBF < 30% and DT > 3 CTP lesion volumes, and 24-h DWI volume. The collateral index was calculated as delay time > 6 volume divided by delay time > 2 volume [7]. In addition to qualitative observations, volumes and ratios observed between ACA, MCA, and PCA territories were recorded for both the ipsilateral and contralateral hemispheres.

### Imaging acquisition protocol

CT imaging in all cases was performed on a Toshiba Aquilion One 320 Slice (Toshiba Medical Systems, Otawara, Japan). A 50 ml bolus of contrast agent (ULTRAVIST 370; Bayer HealthCare, Berlin, Germany) injected in the cubital fossa at a rate of 6 ml/s was used to acquire CTP images. CTP acquisition was performed over a non-contrast mask, automatically commencing 7 s postinjection with scanning at 19-time points (whole brain) over 72 s.

Canon Medical Systems Corporation provided VTM software for use in this study. The VTM technical procedure is described by Matsubara et al. [1] To summarise, seed points are automatically identified via an algorithm immediately distal to the Circle of Willis (A2 segment, bilateral M1 segments, and bilateral P2 segments). Seed points are overlayed onto a TTP (time to peak) map. Using the seed points, a vascular territory is determined by a regional growing method. The regional growing method expands out a vascular territory by finding gradually increasing TTP from the seed point. This is continued until both cerebral hemispheres are mapped.

All perfusion imaging was post-processed on commercial software MiSTAR (Apollo Medical Imaging Technology, Melbourne, Australia). Validated thresholds were applied to measure the volume of the acute perfusion lesion (relative delay time, DT > 3 s) and acute infarct core (relative CBF < 30%). Penumbral volume was calculated from the volume of the perfusion lesion (DT threshold > 3 s) minus the volume of the infarct core (relative CBF threshold < 30% within the DT > 3 s lesion). Occlusion was assessed by true multiphase (dynamic) CTA using all time points acquired during CTP, displayed as maximum intensity projection images.

### Statistical analysis

The statistical analysis was performed using SPSS version 16. Continuous variables were presented as medians with interquartile ranges and categorical variables with frequency and percentages. Although this paper was primarily descriptive, the Mann–Whitney *U* test was performed to establish differences between occlusion types and normal (Supplemental Table 1).

## Results

### Population and demographics

A total of 167 patients were analysed with VTM software. The median age was 73 years [IQR 64–81]. The time from onset of symptoms to scan was a median of 2.1 h [IQR 1.5–3.5] and the median NIHSS was 13 [IQR 8–18]. Other demographic information is listed in Supplemental Table 2. When categorised by occlusion location, there were 59 M1, 44 M2, 5 ACA, 9 PCA, 25 ICA, and 19 with no occlusion.

### Imaging characteristics

Overall, patients with occlusions had a median perfusion core volume of 17 ml [IQR 5–39] and a penumbral volume of 72 ml [IQR 38–100]. Overall, the median 24-h DWI volume was 28 ml [IQR 8.1–70]. Differences between occlusion types are listed in Table 1. VTM map volumes and patterns varied depending on occlusion location and have been summarised by location.

**Table 1:** Imaging variables

Occlusion location	M1	M2	PCA	ACA	ICA	No occlusion
Total (*N*)	59	44	9	5	25	19
CT Perfusion and MRI						
CBF < 30% Median [IQR]	20 [6–41]	11 [3.5–18]	3.5 [0–5.0]	5.0 [2.0–9.0]	48 [30–89]	0 [0–0]
DT < 3 Median [IQR]	119 [86–151]	54 [41–90]	37 [29–46]	35 [34–36]	169 [139–226]	0 [0–0]
Collateral index Median [IQR]	0.26 [0.13–0.34]	0.23 [0.1–0.36]	0.07 [0–0.22]	0.21 [0.19–0.31]	0.34 [0.21–0.46]	0 [0–0]
DWI volume Median [IQR]	20 [8.5–65]	25 [7.5–49]	16 [3.0–33]	5.1 [2.0–20]	94 [43–166]	1 [0–6]
Left hemisphere * N* (%)	27 (46)	26 (59)	5 (56)	3 (60)	13 (52)	n/a
VTM map volumes* (O) = hemispheric side of the occlusion or left hemisphere if no occlusion						
ACA (O) Median [IQR]	241 [193–347]	206 [176–241]	159 [99.1–218]	96 0.1 [40.0–123]	220 [80.8–283]	135 [96–156]
ACA Median [IQR]	132 [90.0–155]	156 [121–191]	159 [151–207]	188 [123–204]	107 [79.5–177]	147 [131–193]
MCA (O) Median [IQR]	58.7 [24.7–98.5]	198 [121–253]	372.9 [313–387]	374 [368–384]	32.2 [15.2–90.9]	351 [324–397]
MCA Median [IQR]	348 [271–361]	304 [255–354]	340 [232–340]	363 [301–418]	307 [253–397]	345 [308–377]
PCA (O) Median [IQR]	302 [268–361]	238 [203–295]	119 [95.5–132]	219 [181–266]	361 [292–401]	183 [161–226]
PCA Median [IQR]	206 [148–247]	196 [145–237]	132 [103–186]	157 [146–158]	214 [157–273]	202 [178–228]

### No occlusion

**Fig. 1:**
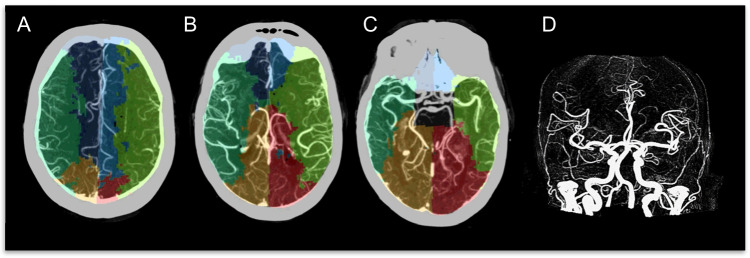
VTM demonstration for a patient without occlusion. Imaging from a patient without vessel occlusion. **A**, **B**, **C** Axial slices of CTA with VTM overlay. **D** CTA MIP frontal view. Each colour defines one of 6 territories: ACA (dark and light blue), MCA (dark and light green), and PCA (orange and red). The VTM software automatically defines seed points on the origin of each major artery (ACA, MCA, and PCA), after which each vascular territory is mapped out. The bolus from these seed points is tracked into the peripheral tissue on a time-to-peak (TTP perfusion) and an algorithm identifies which seed point provides the peak value of TTP to a voxel

Patients without occlusion, as expected, did not have significant CT perfusion abnormalities (median core and perfusion of 0). In this setting, VTM maps typically produce a map with near symmetry of vascular regions (Fig. 1). The median ratio between left and right hemisphere volumes was 1.0 [0.81–1.2]. Median territory volumes (both hemispheres pooled) were ACA: 154 ml [125–193], MCA: 350 ml [322–396], and PCA: 180 ml [151–214].

### Middle cerebral artery occlusion

**Fig. 2:**
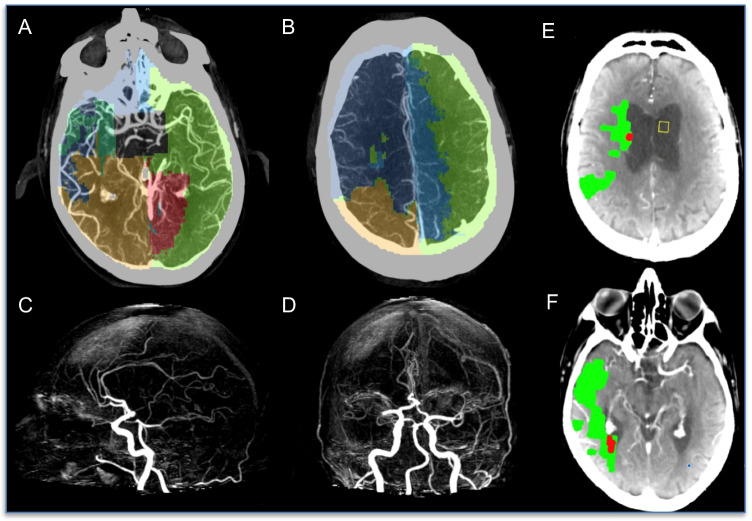
Right MCA occlusion with excellent collaterals. Occluded right M1 segment occlusion. **A**, **B** Axial slices of CTA with VTM overlay. **C**, **D** CTA MIP demonstrates occlusion of the right MCA at the horizontal segment. **E**, **F** MIStar CT Perfusion summary core(red)/penumbra(green) maps (red: CBF < 30% volume, green: DT > 3 volume). VTM software-derived vascular regions show the region supplied by the right MCA is very small (dark green on lower slice A and none on upper slice, B) with a compensatory equal increase of both ACA and PCA regions on the ipsilateral side. The left hemisphere retains the normal VTM pattern. The penumbral region (green) is identified on CT perfusion extending from the right internal capsule to the temporoparietal region. There is minimal perfusion core due to excellent collateralization (demonstrated on both VTM and CTA). On CTP penumbra volume is 83 ml and core is 6 ml. Notably, since collateral flow is very good, some areas of the ischemic MCA territory (supplied by ACA on VTM) have only small delays in contrast to arrival (i.e. delay time < 3 s) and so are under the penumbral threshold (DT > 3 s)

**Fig. 3:**
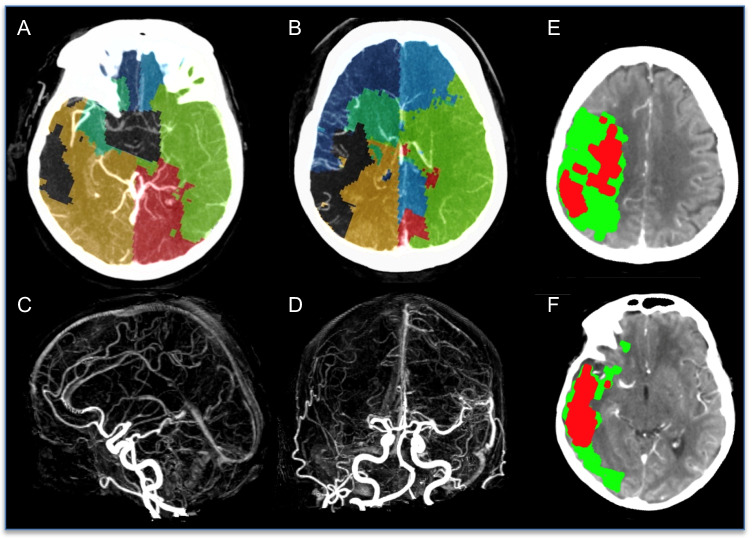
Right MCA occlusion with poor collaterals. Occluded proximal right M1. **A**, **B** Axial slices of CTA with VTM overlay. **C**, **D** MIP CTA demonstrates occlusion of the right MCA. **E**, **F** CT perfusion summary (core/penumbra map) slices. In contrast to the patient in Fig. 2, here is a patient with a poor collateral circulation that demonstrates a minimal supply to the right MCA region (dark green) from the ipsilateral MCA with some compensatory increase of both ACA and PCA supply on the ipsilateral side. Affected hemisphere ACA and PCA territories have not expanded to the same extent as seen in Fig. 2, with smaller ipsilateral ACA/PCA territories on VTM. Given very delayed regions of contrast flow due to this poor retrograde flow from ACA and PCA within the (normally) right MCA supplied territory, there are areas with an absence of territory marking (grey). The penumbral region on CT perfusion extends to the entirety of the right MCA territory (green), again reflecting the marked delay in contrast transit (delay time > 3 s) due to poor collateral flow. On CTP there is 213 ml of penumbra (green) and 78 ml of perfusion core (red). The substantial perfusion core is present in the watershed zones (boundary of ACA/PCA territories) on the VTM map, and is very similar to the unmasked (grey) area on the VTM map, reflecting profound ischemia and severe delay

There were 59 cases with M1 occlusion and 44 with M2 occlusion that was included. In the setting of MCA occlusion, the VTM pattern resulted in a smaller region for the occluded MCA territory and enlargement of ACA and PCA territories in the affected hemisphere. In the setting of M1 occlusion, the median VTM volume of the occluded MCA territory was 58.7 ml [IQR 24.7–98.5], compared to 348 ml [IQR 271–361] in the normal hemisphere. Two typical MCA occlusion VTM maps are displayed: one with excellent collaterals (Fig. 2) and another with poor collaterals (Fig. 3).

### Other occlusion types

**Fig. 4:**
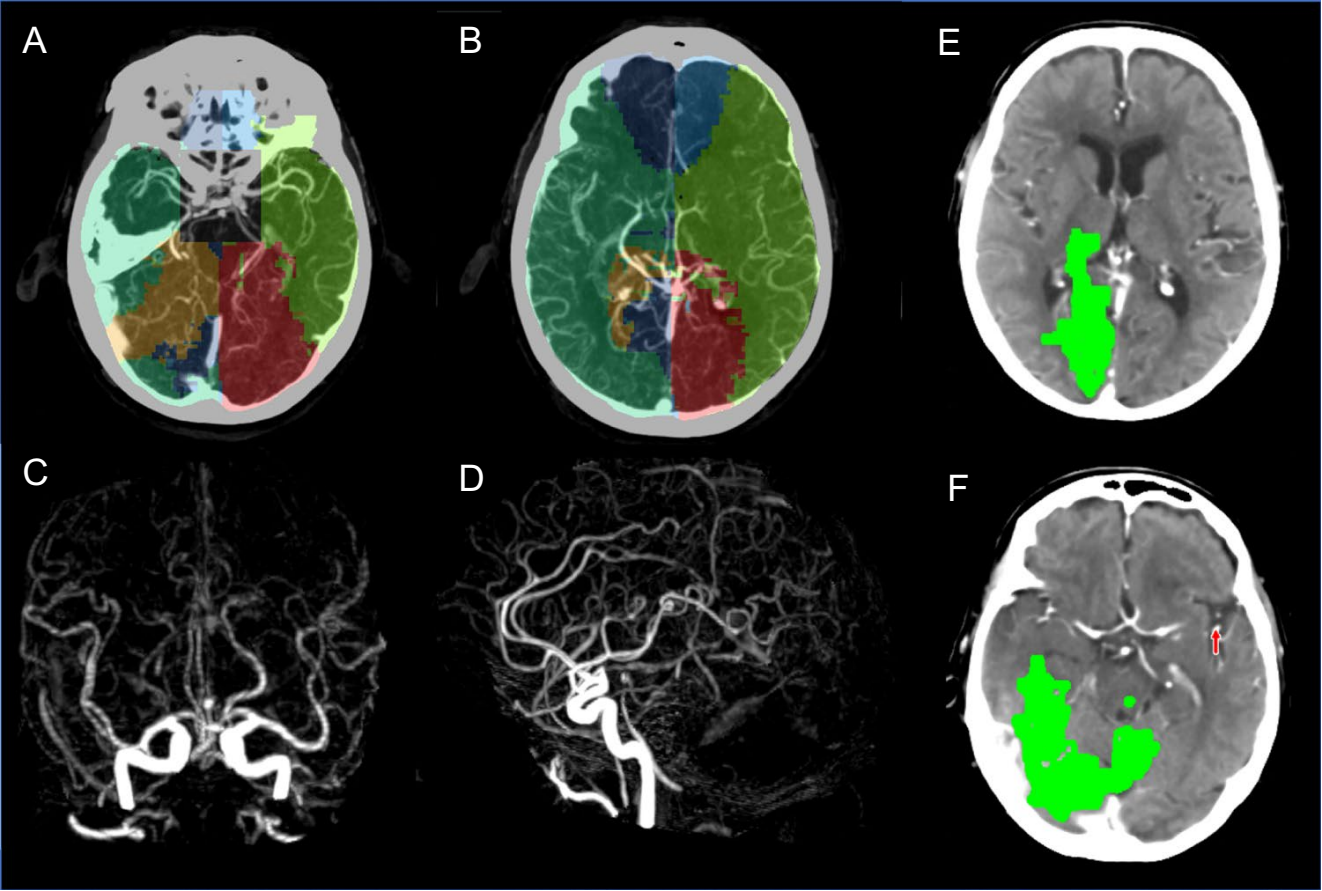
Right PCA occlusion. Occluded pre-communicating segment (P1) of the right PCA. **A**, **B** Axial slices of CTA with VTM overlay. **C**, **D** MIP CTA demonstrates occlusion of the right PCA. **E**, **F** CT Perfusion summary slices. VTM software-derived vascular regions show a reduced right PCA region (yellow) with an increase of both ACA and MCA regions on the ipsilateral side. Notably, the penumbra on CT perfusion within the right PCA territory has a complete absence of CT perfusion core, due to the excellent right MCA collaterals that have been identified on VTM. On CTP there is 141 ml of penumbra (green) and 0 ml of perfusion core (red)

**Fig. 5:**
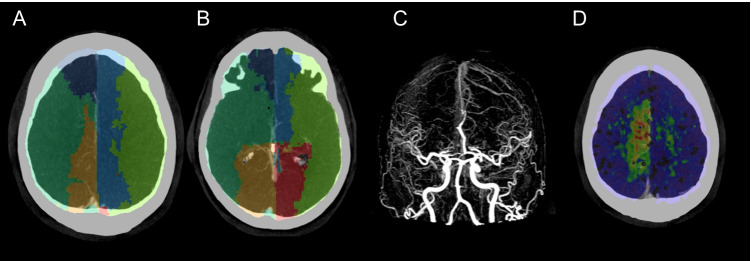
Right ACA occlusion. Occluded A2 segment of the right ACA. **A**, **B** Axial slices of CTA with VTM overlay. **C** MIP CTA demonstrates a hypoplastic right A1 and an absence of distal opacification in the right ACA territory. **D** CT perfusion (Tmax). VTM software-derived vascular regions show a reduced right ACA region (dark blue) with an equal increase of both MCA and PCA regions on the ipsilateral side. CT perfusion demonstrates a penumbral region within the right ACA territory. When correlating CT perfusion with VTM, the delayed region on Tmax is located in the VTM region of ACA that has been replaced by MCA and PCA collaterals

Similar to MCA territory occlusion VTM patterns for ACA, PCA and ICA generated smaller volumes within their corresponding territories. Typical VTM patterns for PCA (Fig. 4) and ACA (Fig. 5) are shown below.

## Discussion

Automated VTM utilising CTP is a novel imaging modality that may have clinical utility in hyperacute stroke assessment. Importantly, VTM provides complementary information to the usual CTP maps without the need for additional imaging. Thus, with further clinical validation, it could be easily incorporated into the current hyperacute stroke imaging assessment workflow without introducing any delay.

Each occlusion location, and indeed every individual patient, demonstrates unique VTM spatial characteristics which are dependent on individual vascular anatomy and collateral flow. As this is a new method, the clinical utility of VTM remains to be tested. However, these examples present multiple points of interest for future validation. For instance, in the setting of MCA occlusion, there is a large increase in ACA and PCA VTM volume compared to the normal (contralateral) side. Expansion of collateral contribution from the non-occluded cerebral vessels is an expected pattern, which VTM was able to confirm. We hypothesise that the volume ratios (such as ACA:PCA volume) may show associations with clinical and imaging outcome measures (DWI volume, collateral failure, functional outcome, etc.). As the VTM process becomes more defined, it could be included in outcome prediction models, further strengthening the selection process for endovascular therapy.

The shortcomings of VTM have not been the focus of the current paper. However, there are downsides to the use of an algorithm-based seeding method. The reported rate of seeding failure using the original algorithm was 16% [1], however even automated CTP is subject to similar flaws (poor arterial input function placement, core overestimation, etc.) [8]. Similar to CTP, physicians will need to be able to identify common artefact patterns so that these errors can be appropriately discarded/rectified. If CTP is unavailable, VTM is unable to be performed using standard CTA due to its need for temporal resolution. Additionally, due to the technical limitations of the seeding process, smaller vessel territories remain difficult to isolate (such as the superior cerebellar artery) and occlusion territories which are interconnected distal to the seeding site (i.e. terminal ICA occlusion) may be complex to interpret. These limitations may be improved in future versions of the automation algorithm.

## Conclusion

Vascular territory mapping provides a quantification of collateral flow/contribution which can be extracted from standard CT perfusion. VTM visually appeared to correlate well with CT perfusion maps, with CT perfusion core matching ‘watershed’ VTM territories where there was a severe delay between vascular territories. Although CT perfusion provides an overall estimation of core and perfusion volume, territory mapping further builds a qualitative and quantitative map of vessel-specific contributions. These vessel-specific contributions may have management and prognostic implications. Further studies are required to define its role.

## Supplementary Information

Below is the link to the electronic supplementary material.

Supplementary file1 (DOCX 17 KB)

## Abbreviations

VTM: Vascular territory mapping
NCCT: Non-contrast computerized tomography
CTA: Computerised tomography arch to vertex angiogram
CTP: Computerised tomography perfusion
LVO: Large vessel occlusion
ACA: Anterior cerebral artery
MCA: Middle cerebral artery
M1: 1St segment middle cerebral artery
M2: 2Nd segment middle cerebral artery
PCA: Posterior cerebral artery
ICA: Internal carotid artery
TTP: Time to peak
CBF: Cerebral blood flow
DT: Delay time
DWI: Diffusion-weighted imaging
MRI: Magnetic resonance imaging
NIHSS: National Institutes of Health Stroke Scale
SPSS: Statistical Package for the Social Sciences
IQR: Interquartile range
Tmax: Time to maximum
MIP: Maximum intensity projection
