# Supplementary material for: CT vascular territory mapping: a novel method to identify large vessel occlusion collateral
Source: Neuroradiology. 2022 Aug 11;65(1):113–9. doi: 10.1007/s00234-022-03034-4 (PMC9816260; doi:10.1007/s00234-022-03034-4)
Supplement: Supplementary file 1 — Supplementary file1 (DOCX 17 KB) [file 234_2022_3034_MOESM1_ESM.docx]

**Supplementary information**

**Supplemental Table One: Mann-Whitney U tests**

|  | **M1**  **OCCLUSION** | **NO OCCLUSION** | **P VALUE** |
| --- | --- | --- | --- |
| ACA (O),  median [IQR] | 241  [193-347] | 147  [131-193] | 0.001 |
| ACA,  median [IQR] | 132  [90.0-155] | 135  [96-156] | 0.150 |
| MCA (O),  median [IQR] | 58.7  [24.7-98.5] | 345  [308-377] | 0.001 |
| MCA,  median [IQR] | 348  [271-361] | 351  [324-397] | 0.676 |
| PCA (O),  median [IQR] | 302  [268-361] | 202  [178-228] | 0.001 |
| PCA,  median [IQR] | 206  [148-247] | 183  [161-226] | 0.956 |

|  | **M2**  **OCCLUSION** | **NO OCCLUSION** | **P VALUE** |
| --- | --- | --- | --- |
| ACA (O),  median [IQR] | 206  [176-241] | 147  [131-193] | 0.001 |
| ACA,  median [IQR] | 156  [121-191] | 135  [96-156] | 0.214 |
| MCA (O),  median [IQR] | 192  [121-253] | 345  [308-377] | 0.001 |
| MCA,  median [IQR] | 304  [255-354] | 351  [324-397] | 0.002 |
| PCA (O),  median [IQR] | 238  [203-295] | 202  [178-228] | 0.001 |
| PCA,  median [IQR] | 196  [145-237] | 183  [161-226] | 0.679 |

|  | **PCA**  **OCCLUSION** | **NO OCCLUSION** | **P VALUE** |
| --- | --- | --- | --- |
| ACA (O),  median [IQR] | 162  [110-218] | 147  [131-193] | 0.425 |
| ACA,  median [IQR] | 155  [151-206] | 135  [96-156] | 0.043 |
| MCA (O),  median [IQR] | 361  [324-385] | 345  [308-377] | 0.871 |
| MCA,  median [IQR] | 340  [272-340] | 351  [324-397] | 0.058 |
| PCA (O),  median [IQR] | 120  [104-142] | 202  [178-228] | 0.001 |
| PCA,  median [IQR] | 134  [114-209] | 183  [161-226] | 0.013 |

|  | **ACA**  **OCCLUSION** | **NO OCCLUSION** | **P VALUE** |
| --- | --- | --- | --- |
| ACA (O),  median [IQR] | 96 .1  [40.-123] | 147  [131-193] | 0.075 |
| ACA,  median [IQR] | 188  [123-204] | 135  [96-156] | 0.344 |
| MCA (O),  median [IQR] | 374  [368-384] | 345  [308-377] | 0.081 |
| MCA,  median [IQR] | 363  [301-418] | 351  [324-397] | 0.762 |
| PCA (O),  median [IQR] | 219  [181-266] | 202  [178-228] | 0.544 |
| PCA,  median [IQR] | 157  [146-158] | 183  [161-226] | 0.058 |

|  | **ICA**  **OCCLUSION** | **NO OCCLUSION** | **P VALUE** |
| --- | --- | --- | --- |
| ACA (O),  median [IQR] | 220  [86.8-281] | 147  [131-193] | 0.05 |
| ACA,  median [IQR] | 107  [82.7-159] | 135  [96-156] | 0.146 |
| MCA (O),  median [IQR] | 32.2  [19 - 159] | 345  [308-377] | 0.001 |
| MCA,  median [IQR] | 307  [254-376] | 351  [324-397] | 0.211 |
| PCA (O),  median [IQR] | 361  [307-396] | 202  [178-228] | 0.001 |
| PCA,  median [IQR] | 214  [165-267] | 183  [161-226] | 0.461 |

| **Supplemental Table Two: Demographics** | | | | | | |
| --- | --- | --- | --- | --- | --- | --- |
| **OCCLUSION LOCATION** | **M1** | **M2** | **PCA** | **ACA** | **ICA** | **NO OCCLUSION** |
| Total, N | 59 | 44 | 9 | 5 | 25 | 19 |
| Age, median [IQR] | 73  [64-79] | 79  [70-83] | 83  [74-88] | 80  [61-83] | 65  [57-75] | 69  [49-74] |
| Males, N (%) | 34 | 15 | 4 | 4 | 16 | 12 |
| NIHSS, median [IQR] | 17  [13-19] | 11  [6-15] | 9  [6-12] | 6  [5-8] | 17  [15-18] | 6  [2-11] |
| Glucose, median [IQR] | 7  [6-8] | 7  [6-8] | 8  [6-9] | 7  [7-8] | 7  [6-8] | 6  [6-7] |
| Blood pressure, median | 140/80 | 158/86 | 179/98 | 143/82 | 140/81 | 150/82 |
| Onset to scan (hours), median [IQR] | 1.7  [1.5-2.6] | 2.1  [1.5-4.6] | 2.4  [2.1-3.3] | 3.2  [2.5-5.6] | 1.9  [1.3-3.7] | 2.4  [1.7-4.7] |
| Anticoagulation, N(%) | 7 (12) | 5 (11) | 0 (0) | 1 (20) | 1 (4) | 2 (11) |
| Co-morbidities | | | | | | |
| Smoking, N(%) | 9 (15) | 3 (7) | 0 (0) | 0 (0) | 6 (24) | 3 (16) |
| Hypertension, N(%) | 38 (64) | 28 (64) | 7 (78) | 1 (20) | 11 (44) | 9 (47) |
| Atrial Fibrillation, N(%) | 21 (36) | 19 (43) | 2 (22) | 1 (20) | 7 (28) | 4 (21) |
| Hyperlipidemia, N(%) | 18 (31) | 17 (39) | 4 (44) | 2 (40) | 6 (24) | 6 (32) |
| Diabetes, N(%) | 9 (15) | 7 (16) | 1 (11) | 2 (40) | 3 (12) | 0 (0) |
| Transient ischaemic attack, N(%) | 6 (10) | 6 (14) | 1 (11) | 1 (20) | 2 (8) | 2 (11) |
| Previous ischaemic stroke, N(%) | 6 (10) | 6 (14) | 1 (11) | 0 (0) | 2 (8) | 2 (11) |
